# Controlled Release of Perillyl Alcohol via pH-Responsive Chitosan-Polypyrrole Nanocarriers

**DOI:** 10.1021/acsomega.5c04817

**Published:** 2025-06-25

**Authors:** Marcella Matos Cordeiro Borges, Stephanne Yonara Barbosa de Carvalho, Keyller Bastos Borges, Luiz Gustavo de Lima Guimarães

**Affiliations:** Departamento de Ciências Naturais, 74383 Universidade Federal de São João del-Rei, Campus Dom Bosco, Praça Dom Helvécio 74, Fábricas, 36301-160 São João del-Rei, Minas Gerais, Brazil

## Abstract

This work focused on developing CS/PPy, a pH-responsive controlled-release nanocarrier composed of natural polymer, the chitosan (CS), and synthetic polymer, the polypyrrole (PPy). The goal was to evaluate its potential application in controlled release studies of perillyl alcohol (POH). To enhance its properties, CS/PPy was cross-linked with glutaraldehyde, resulting in a new material, CS/PPy/GA. CS/PPy and CS/PPy/GA demonstrated high encapsulation efficiency for POH. The release of POH was investigated at pH 4.5, 6.0, and 7.4. The POH release rate exhibited pH-dependent behavior, with a significantly higher release observed in acidic media (pH 4.5) compared to neutral/basic media (pH 7.4). This suggests the potential for controlled release of POH in response to specific physiological environments. The Korsmeyer-Peppas model was followed for the release of POH from CS/PPy at all pH values that were studied. The release behavior of POH from CS/PPy/GA exhibited a pH-dependent mechanism. The Korsmeyer-Peppas model best described release at pH 4.5 and 6.0, while the Higuchi model provided a better fit at pH 7.4. The results suggested that the synthesized materials exhibited high affinity for POH, and the release behavior is pH-dependent.

## Graphical abstract

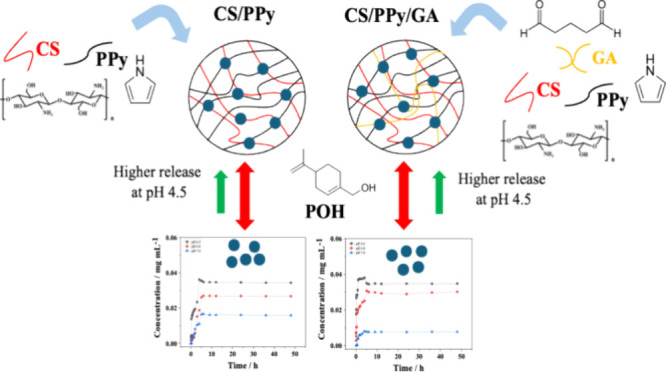

## 1 Introduction

Cancer, a disease that inspires significant fear globally, remains a leading cause of mortality. Are the estimated ten million deaths attributed to cancer in 2020, with millions of new cases continuing to emerge annually.^1^ Cancer is characterized as a complex disease due a multitude of significant genetic alterations, leading to uncontrolled cell proliferation and cell death. Surgery, chemotherapy, and radiotherapy represent the cornerstone of conventional cancer treatment.^2^

Therapeutic agents considered effective for treating cancer exhibit side effects due to their toxicity, often limiting the dose to be delivered and limiting the effectiveness of the treatment. These drugs, while effective at targeting cancer cells, can also harm healthy tissues and organs due to unintended cytotoxicity, leading to collateral cell death. In this context, research is actively seeking less-toxic alternatives with anticancer activity, such as those derived from natural sources molecules.^3^

Peryllyl alcohol (POH) is a monocyclic terpene derived from limonene. These compounds integrate a diverse class of natural substances known as monoterpenes, promising for their antioxidant and antitumor properties. POH can be considered an amphiphilic substance, demonstrating limited water solubility due to its lipophilic properties. This amphiphilic nature allows POH to readily cross cell membranes and potentially the blood-brain barrier, facilitating its therapeutic action or interaction with other materials which it is associated.^4^ POH exhibits promising chemopreventive activity; however, its high volatility and low solubility in aqueous pose significant hurdles for practical application.^4^ In view of this, new strategies that can improve the administration of drugs targeted to cancer cells and that do not cause side effects in healthy cells have been outlined. Controlled release nanocarriers offer a promising option to minimize adverse reactions to chemotherapy drugs by delivering them directly to the target site. These nanocarriers excel at encapsulating bioactive therapeutic molecules. This encapsulation offers benefit such as reduction the drug’s toxic effects on healthy tissues, protection it from degradation within the body, and allows for gradual transport and targeted delivery to target cells. The carrier nanoparticles pass through the permeable blood vessels responsible for nourishing growing tumors, enter the cell more easily and accumulate within the solid tumor.^5^

Polymeric biomaterials like CS are gaining significant attention for their potential as drug nanocarriers due to their biocompatibility and the presence of chemical functional groups within their structure, allowing for specific functionalities. Combining two or more polymers, exemplified by the coupling of a natural polymer, as chitosan, with synthetic polymers, as polypyrrole (PPy), offers a strategy to enhance the physicochemical properties of controlled release nanocarriers.^6^ CS/PPy composites have emerged as efficient platforms for drug delivery in nanomedicine due to their impressive combination of properties. These composites exhibit desirable characteristics for controlled release, including mechanical strength, redox activity, biocompatibility, biodegradability, stability, adjustable conductivity, and the ability for rapid and reversible changes in oxidation state (doping).^7,8^

A biodegradable composite based on conductive nanoparticles using PPy and CS has been developed to explore their potential as carriers for 1,2,4-triazoles.^9^ Significant strides in thrombosis treatment by developing PPy nanoparticles embedded with CS glycol to enhance heparin delivery and achieve therapeutic efficacy were archived. This study demonstrated a significant improvement in fibrin clot clearance. This success can be attributed to the developed nanocarrier, which facilitated enhanced heparin delivery to the thrombus, leading to a shorter treatment time.^10^ CS/PPy nanocomposite has been also formulated and evaluated specifically for controlled release of the anticancer drug doxorubicin.^7^ Another work consisted in synthesis of an electroactive polyacrylamide/CS/PPy hydrogel for controlled release of captopril.^11^ These findings strongly support the development of polymeric materials based on CS and PPy for controlled release systems, showing their versatility and applications potential.

Therefore, this work presents the development of innovative pH-responsive nanocarriers based on CS coupled to PPy. Because the two components work in concert to improve the adsorption property, the chitosan-pyrrole system was selected. The polypyrrole has weak mechanical characteristics and is insoluble. Chitosan might enhance these qualities. These nanocarriers demonstrate the ability to encapsulate and release POH in a controlled manner within aqueous environments. The synthesized nanocarriers were properly characterized by scanning electron microscopy coupled to an energy dispersive spectrometer (SEM/EDS), transmission electron microscopy (TEM), infrared spectroscopy (FTIR), thermogravimetric analysis (TGA), X-ray diffraction (XRD), Dynamic light scattering (DLS), surface wettability analysis, and zeta potential (pH_PZC_) measurements. Following the characterizations, the nanocarriers was investigated to controlled release of POH.

## 2 Experimental Section

### 2.1 Solvents, Reagents, Standard and Stock Solutions

Pyrrole (98%), CS (medium molecular weight and degree of deacetylation of 75–85%), sodium dodecyl sulfate (SDS, ≥98.5%), POH (96%), and ammonium persulfate (APS) were purchased from Sigma-Aldrich (St. Louis, MO, USA). Glutaraldehyde (GA), sodium hydroxide (NaOH, 97%) was obtained from Dinâmica (Diadema, SP, Brazil). Acetic acid, hydrochloric acid, phosphoric acid (85%), and boric acid were purchased from Synth (Diadema, SP, Brazil). Toluene and methanol were obtained from Vetec (Rio de Janeiro, RJ, Brazil) and ethanol purchased from Cinética (Itapevi, SP, Brazil). Ultrapure water was purified and distilled using the Millipore Milli-Q Plus system (Bedford, Massachusetts, USA).

### 2.2 Apparatus and Experimental Conditions

Particle size distribution was measured using a DelsaNano C particle analyzer (Beckman Coulter, Brea, CA, USA). Encapsulation efficiency (EE%) of POH and the tests of in vitro release were determined using a UV-2550 model diode array spectrophotometer (Shimadzu, Tokyo, Japan). Measurements were performed at room temperature (25 °C) using a quartz cuvette (Hellma). The wavelength of maximum absorption (λ_max_) of POH was determined by UV–vis spectroscopy, scanning from 190 to 400 nm. The morphology of the synthesized materials was investigated using a Hitachi Analytical Tabletop TM3000 scanning electron microscope equipped with energy-dispersive X-ray spectroscopy (SEM/EDS) (Tarrytown, NY, USA). The samples were applied on carbon tape with an acceleration voltage of 20 kV. Transmission electron microscopy (TEM) images were obtained using a JEOL JEM-1400 microscope (Denton Vacuum, Moorestown, NJ, USA) at various magnifications between 20 and 100 nm. The FTIR analyses for all materials were obtained in the 4000–400 cm^–1^ region using the conventional method of KBr tablet by FTIR spectrometer (Spectrum GX PerkinElmer). A Shimadzu DTG-60H thermobalance (Japan) was used to obtain thermogravimetric analysis (TGA) data under nitrogen flow (50 mL min^–1^) at a heating rate of 10 °C min^–1^ from 25 to 1000 °C. The XRD analyses were performed by a Vinci Advance-Bruker Diffractometer-D8 operating with radiation: Cu-kα1 = 1.54059 Å and kα2 = 1.54443 Å. To evaluate the wettability, i.e., the evaluation of the hydrophobicity/hydrophilicity of the materials, a Nikon model D90 camera equipped with a 50 mm Nikon lens was used to provide high-resolution images.

### 2.3 Synthesis of pH-Responsive and Magnetic Nanocarriers

#### 2.3.1 Synthesis of CS/PPy

A solution of 150 mg CS in 1% (v/v) acetic acid (15 mL) was stirred magnetically at 1000 rpm using an IKA C-MAG HS-7 shaker for 24 h at 25 °C. To a beaker containing 15 mL of 1 M HCl, 321 μL of pyrrole were added. In another beaker, 531 mg of APS were dissolved in 7.5 mL of HCl (1 mol L^–1^). The solutions from both beakers were then simultaneously added dropwise to the stirred CS solution under constant magnetic stirring. The combined solution was stirred for 1 h in an ice bath under light exclusion. Following the ice bath treatment, the mixture was stirred continuously for an additional 24 h at room temperature (25 ± 3 °C) under light exclusion. After, ethanol (70 mL) was added to induce precipitation of the synthesized nanocarriers. The precipitate was recovered by centrifugation and subsequently washed with ethanol using sonication for 10 min (repeated five times) to eliminate unreacted pyrrole and other residual reagents. Finally, the material was dried at 60 °C for 24 h.^12^

#### 2.3.2 Synthesis of CS/PPy/GA

The cross-linking of the CS/PPy precursor material was adapted from literature with some modifications.^12^ Thus, 100 mg of the previously synthesized CS/PPy nanoparticles were dispersed in 10 mL of 1 M HCl. Next, 100 μL of GA and a solution containing 0.173 g of SDS in 10 mL of toluene were added. This mixture was sonicated in an ice bath for 10 min. Subsequently, the ice bath was removed, and the reaction continued for another 24 h at room temperature (25 ± 3 °C). The final product was then centrifuged at 5000 rpm, redispersed in 10 mL of ultrapure water, and dialyzed against ultrapure water using a 14.000 MWCO membrane for 3 days to remove unreacted salts and monomers. Finally, the material was dried at 60 °C for 24 h.

### 2.4 pH_PZC_ and Wettability

The isoelectric point (IEP), also known as the point of zero charge (pH_PZC_), of each synthesized material was experimentally determined. This involved adjusting the pH of aqueous dispersions of the materials to 2.04, 4.02, 6.05, 8.09, and 10.05 using 0.1 M NaOH and 0.1 M HCl. A quantity of 10 mg of each material was placed in contact with 7 mL of each solution in Falcon tubes. The tubes were vortexed for 1 min at 3000 rpm and room temperature (25 ± 3 °C). After this time, they were left to rest for 24 h. Following the 24 h equilibration period, the final pH of each suspension was measured. The experiment was performed in triplicate for each material. The pH_PZC_ was determined by plotting the initial pH (pH_initial_) versus the final pH (pH_final_) after equilibration. The pH_PZC_ corresponds to the pH value where the initial and final pH values are equal or very close.^13^ The wettability of each material was assessed by placing a small sample on a Petri dish. A drop of water was then deposited on the sample surface, and the resulting contact angle was captured using a Nikon D90 camera equipped with a 50 mm lens.

### 2.5 DLS

The hydrodynamic diameter and size distribution of CS/PPy and CS/PPy/GA, nanoparticles were determined by DLS in Britton-Robinson buffer at pH 7.4, 6.0, and 4.5. To this end, 30 mg of each synthesized material was dispersed in 3 mL of each buffer solution under magnetic stirring for 24 h. Particle size distribution measurements were carried out at room temperature (25 ± 3 °C).

### 2.6 Dispersity Tests

Dispersity tests were carried out for the materials to observe and determine which of these materials presents the best response in acidic, neutral and basic pH environments, which simulate the blood, tumor and lysosomal environment of cells. Initially, three Britton-Robinson buffer solutions were prepared with the aim of simulating the pH of the blood (7.4, 0.006 M), tumor (6.0, 0.004 M) and lysosomal (4.5, 0.003 M) environment of the cells. Then, 5 mg of each material was dispersed in 4.95 mL aliquots of each Britton-Robinson buffer solution using magnetic stirring (1000 rpm) for 24 h at room temperature (25 ± 3 °C). After stirring, the dispersions were centrifuged at 9000 rpm for 10 min using a Sigma 2–16P benchtop centrifuge. Then, 2.5 mL of the supernatant was added to a previously weighed aluminum container (∼2.5 g), which was taken to the oven for the drying process at 100 °C for 24 h. Following oven drying at 100 °C for 24 h, the weight of the dried material in each container was measured. This weight was then used to calculate the solubility of the material in each buffer solution.^14^ The procedure was carried out in triplicate for each sample at different pHs.

### 2.7 POH Encapsulation

The two synthesized polymeric materials (CS/PPy and CS/PPy/GA) were used to encapsulate POH. 50 mg of each material was dispersed in 5 mL of 1% (v/v) acetic acid solution using magnetic stirring for 12 h at room temperature (25 ± 3 °C). Afterward, 50 mg of POH, dissolved in a minimal amount of ethanol (three drops), was slowly added to the stirred polymer dispersions. The dispersions were then sonicated in an ultrasonic bath at 40 kHz for 10 min at room temperature (25 ± 3 °C). While stirring the dispersions, 1 M NaOH was gradually added to increase the pH to 8.5–9.0, triggering the precipitation of materials with encapsulated POH. Once this was done, the mixture was placed in a refrigerator at 4 °C for 24 h. Following the 24-h storage, the dispersions were centrifuged at 9000 rpm for 5 min. The initial supernatant, containing unencapsulated POH, was collected for further analysis to determine the encapsulation efficiency (EE%). Then, successive washes were carried out with ultrapure water until neutral pH was obtained. After discarding the final supernatant (washing waste), the materials with encapsulated POH were transferred to a Teflon plate for drying and stored in a desiccator. All assays were performed in triplicate.^15^

### 2.8 Encapsulation Efficiency (EE%)

The EE% of POH within CS/PPy and CS/PPy/GA was determined using UV–visible spectrophotometry. The analysis employed a UV-2550 spectrophotometer and quartz cuvettes with a 10 mm optical path length. To determine EE%, a 2.5 mL aliquot of the initial supernatant, collected during encapsulation, was diluted in a 10 mL volumetric flask in methanol. The diluted solution was then analyzed using a UV–visible spectrophotometer at a wavelength of 203 nm. The percentage of encapsulated POH in each material was determined by comparing the absorbance of the samples at 203 nm with the previously established calibration curve for POH (Figure S1). Therefore, EE% was calculated according to eq 1.^16^

$$\%\mathrm{EE}=\frac{M_{f}}{M_{i}}\times 100$$ 1

where *M*_i_ represents the initial mass of POH added during encapsulation, and *M*_f_ represents the mass of free (unencapsulated) POH remaining in the supernatant after the encapsulation process.

### 2.9 In Vitro Release Profile

The in vitro release profiles of POH by the synthesized materials were investigated in Britton-Robinson buffers at pH 7.4 (0.006 M), 6.0 (0.004 M), and 4.5 (0.003 M).^17^ To investigate the release behavior, 30 mg of material with encapsulated POH was dispersed in 2 mL of the corresponding Britton-Robinson buffer (pH 7.4, 6.0, or 4.5) and transferred into a 14.000 MWCO dialysis membrane. The dialysis membrane was pretreated by soaking it in the corresponding buffer solution (pH 7.4, 6.0, or 4.5) for 8 h before being filled and sealed. The sealed dialysis membrane, containing the material, was then immersed in 58 mL of buffer solution (pH 7.4, 6.0, or 4.5) and taken to an incubator (TECNAL, model TE-424) set at 37 °C with constant agitation at 140 rpm. At predetermined time intervals (0.02, 0.05, 0.08, 0.16, 0.25, 0.50, 0.75, 1.0, 1.5, 2.0, 3.0, 4.0, 5.0, 6.0, 9.0, 12.0, 24.0, 36.0, 48.0 h), 1 mL aliquots of the release medium were withdrawn from the incubator. To maintain a constant volume, 1 mL of buffer solution (corresponding pH) was immediately added after each sample collection. The amount of released POH was quantified by UV–visible spectrophotometry using quartz cuvettes with a 10 mm optical path length. The analysis scanned the wavelength range of 190–400 nm. Before analysis, the samples were diluted in 1 mL of methanol. The concentration of POH released in each sample was determined by measuring the absorbance at 203 nm and referencing a previously established calibration curve for POH (Figure S2).

## 3 Results and Discussion

### 3.1 Development and Characterizations of Synthesized Materials

In the process of material synthesis CS/PPy and CS/PPy/GA, in acidic aqueous media (low concentration of acetic acid), the primary amine groups (−NH_2_) of the CS structure become fully protonated (−NH_3_^+^), resulting in a positively charged biopolymer. Acetic acid can induce the structural transformation of CS into oligomers or even monomers, however, this depends on the reaction conditions employed. Under these conditions, the hydrolysis of CS is relatively slow and limited, with no significant degradation occurring for oligomers or monomers. The principal function of acetic acid was to protonate the free amino groups of CS, favoring its solubilization without causing significant degradation of the polymer chain. Some care is necessary, since more severe conditions, such as high concentrations of acetic acid, elevated temperature, prolonged incubation times, or the presence of catalysts, partial hydrolysis of the glycosidic bonds takes place, leading to the formation of CS oligomers and the release of monomers.^7,11^

The thermal dissociation of APS generates sulfate radical anions (SO_4_•^–^) that can initiate the polymerization process. These radicals attack hydrogen or nitrogen atoms in CS chains, forming macro radicals which subsequently react with pyrrole monomers, leading to the growth of the CS/PPy polymer chain. The CS/PPy/GA material formation follows a similar process, but with the addition of GA which cross-links the entire polymer chain. Cross-linking polymer chains enhances their stability and mechanical properties, improving resistance to heat and solvents. While GA exhibits cytotoxicity, its widespread use as a cross-linking agent stems from two key factors: (1) its reaction with amino or hydroxyl groups is rapid due to the presence of aldehyde groups in its structure, and (2) its cytotoxicity is dependent on the concentration.^18^

### 3.2 FTIR

Fourier-transform infrared (FTIR) spectroscopy (Figure 1A) reveals characteristic signals corresponding to the functional groups present in each material. The FTIR spectra of both materials exhibit a high degree of similarity, suggesting the presence of common functional groups. Broad absorption bands can be observed between 3421–3120 cm^–1^ relative to the stretching vibrations of the O–H bond of CS and which appear superimposed on the N–H stretching vibrations of pyrrole. The observed vibrational frequencies suggest hydrogen bonding between the hydroxyl and amine groups of CS and PPy. The bands observed in the region between 2917–2850 cm^–1^ are attributed to the symmetric and asymmetric stretching of the C–H bonds, present in CS. The bands around 1521 and 1411 cm^–1^ correspond to the stretching vibrations of the C=C and C–N bonds of the pyrrole ring. At 1616 cm^–1^ the absorption band is due to the stretching of the C=O bond referring to the carbonyl of the amide group (amide I) in the CS. The absorption bands at 1060, 1008, and 852 cm^–1^ correspond to C–O and C–O–C stretching vibrations in the pyranosidic ring of CS (Figure 1B).^19,20^ The absorption band in the range of 788–804 cm^–1^ indicates the presence of–NH–bonds, proving the formation of CS-based materials linked to PPy. The CS/PPy/GA spectrum reveals a small band around 1718 cm^–1^, indicating the presence of the −HC=N– functional group. This group is characteristic of Schiff base linkages, which can form between amine groups of CS and aldehyde groups of GA during cross-linking.^21^

**Figure 1:**
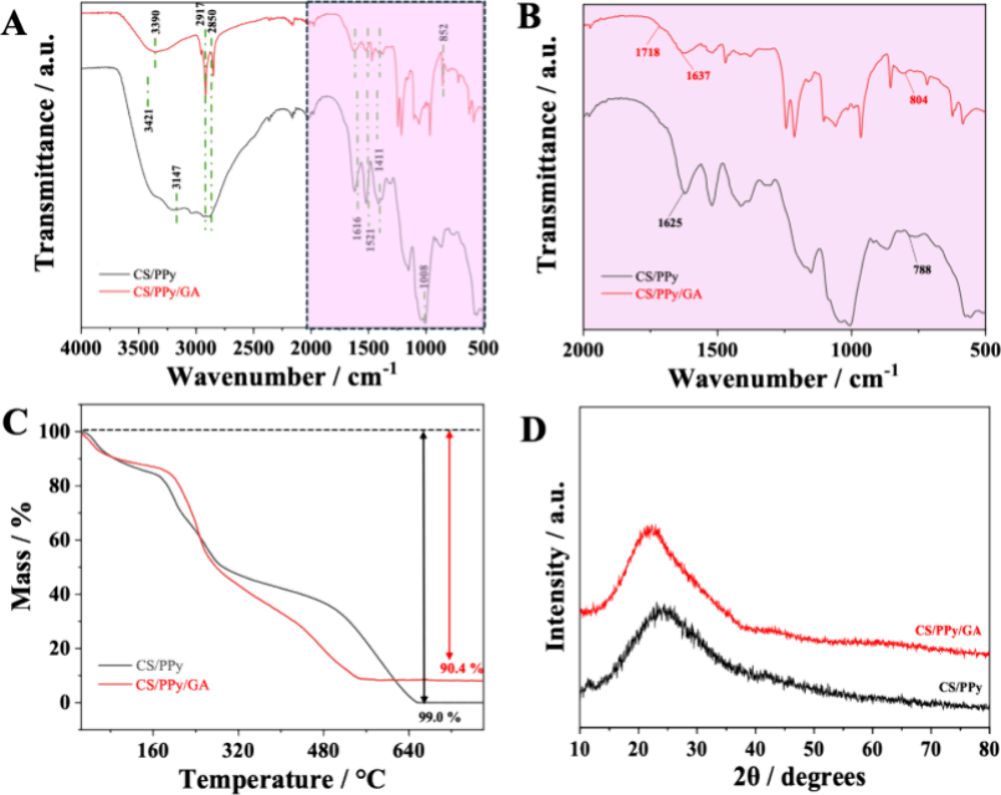
(A, B) FTIR, (C) TGA, and (D) XRD of CS/PPy and CS/PPy/GA.

### 3.3 TGA

The thermal behavior of the materials was determined by TGA, as shown in Figure 1C. CS/PPy and CS/PPy/GA exhibit an initial mass loss below 100 °C, likely due to the removal of moisture and residual volatile compounds from the synthesis process. The CS/PPy curve exhibits a significant mass loss (99%) by the end of the analysis (1000 °C). This mass loss can be attributed to two main stages: degradation of CS between 180–400 °C and degradation of the PPy backbone between 400–550 °C. The TGA analysis suggests that CS/PPy exhibits thermal stability up to around 150 °C. Beyond this temperature, a significant mass loss is observed, indicating the onset of polymer chain degradation. CS/PPy/GA exhibits a similar mass loss pattern, reaching a final mass loss of 90.36% at 1000 °C. The high mass loss is likely due to the predominantly organic nature of these materials.^22,23^

### 3.4 XRD

The crystal structures of the synthesized materials were evaluated by XRD. The diffractograms of CS/PPy and CS/PPy/GA in Figure 1D show broad peaks, suggesting low crystallinity in these materials. The diffractograms show a weak peak at 2θ ≈ 11° characteristic of amorphous CS and a broader peak at 2θ ≈ 25° associated with amorphous PPy.^24^

### 3.5 SEM/EDS

SEM analysis was performed to investigate the morphology of CS/PPy and CS/PPy/GA. The obtained images at magnifications of 500× and 1000× are shown in Figure 2. Both materials exhibited an undefined morphology with a heterogeneous size distribution and an irregular, rough surface. CS/PPy/GA presents a different morphology on the surface of the material, as can be seen in the white arrows.

**Figure 2:**
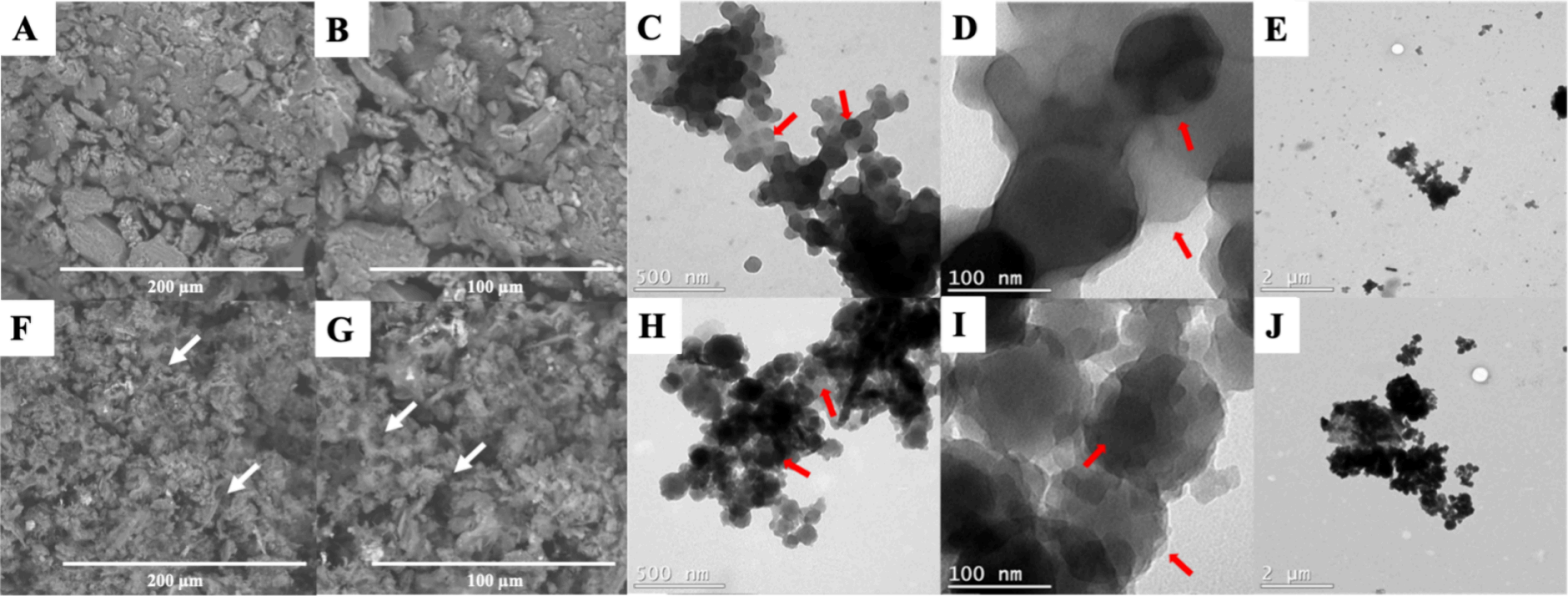
SEM images of (A, B) CS/PPy at magnifications of 500× and 1000× and TEM images of (C-E) CS/PPy at three magnifications (500 nm, 100 nm, and 2 μm); SEM images of (F,G) CS/PPy/GA at magnifications of 500× and 1000×and TEM images of (H-J) CS/PPy/GA, at three magnifications (500 nm, 100 nm, and 2 μm).

In addition, EDS analysis was performed to determine the semiquantitative elemental composition of CS/PPy and CS/PPy/GA. The results are described in Table 1. As expected, elements C and N are present in all materials, as they come from CS and PPy chains. EDS analysis confirms the presence of key elements in CS/PPy and CS/PPy/GA, which is consistent with the successful synthesis of these materials.

**Table 1:** Elementary Composition of Synthesized Materials Obtained by EDS^a^

	elements/%			
materials	C	N	O	impurities^a^
CS/PPy	46.04	12.0	39.48	2.48
CS/PPy/GA	59.09	7.69	31.24	1.98

a Other elements identified.

### 3.6 TEM

The morphology and microstructure of the materials were also investigated using high-resolution transmission electron microscopy (TEM) images (Figure 2). The micrographs reveal that both materials consist of agglomerated, interconnected spherical nanoparticles. TEM images of CS/PPy (Figure 2C–E) reveal a contrast in brightness between regions, indicated by red arrows. The dark spheres are consistent with PPy nanoparticles surrounded by a lighter contrast region, likely corresponding to the CS matrix.^19^ The particles varied in size from 78 to 184 nm. From Figure 2H–J after cross-linking CS/PPy with GA, the spherical morphology of the CS/PPy/GA material is preserved. Compared to CS/PPy, the CS/PPy/GA composite exhibits an increase in the average diameter of the spherical nanoparticles, ranging from 106 to 257 nm. Particle diameters were estimated by measuring nonaggregated, well-dispersed spherical features in the TEM images.

### 3.7 pH_PZC_

The pH_PZC_ is a crucial property that governs the net surface charge of a material in aqueous environments. It significantly influences the adsorption behavior, stability, and colloidal interactions of the material. The Figure 3 shows the pH_PZC_ for the CS/PPy and CS/PPy/GA, which were determined by a graph of pH_initial_ × pH_final_.

**Figure 3:**
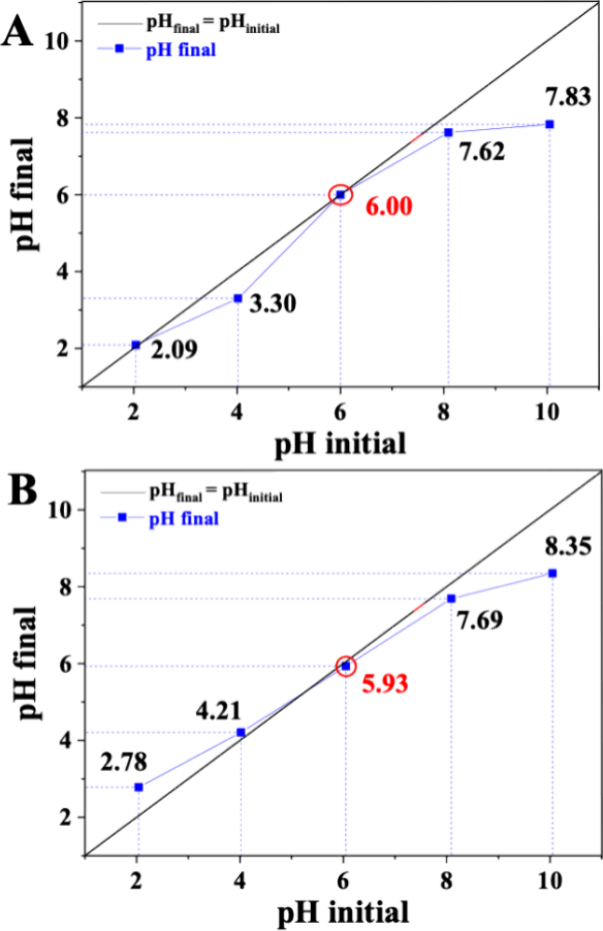
pH_PZC_ of the synthesized materials: (A) CS/PPy and (B) CS/PPy/GA.

The pH_PZC_ values for CS/PPy and CS/PPy/GA were 6.00 and 5.93, respectively. These results suggest minimal changes in net surface charge upon incorporation of GA into the composite. This indicates that at their respective pH_PZC_ values, the net surface charges of CS/PPy and CS/PPy/GA are close to zero. In other words, the materials are near their IEP, where positive and negative charges balance.^25^ The pH_PZC_ values for both materials were found to be close to 6.0, indicating similar net surface charges at this pH. Based on the determined pH_PZC_ values (around 6.0), we can predict that the surfaces of these materials will be positively charged in aqueous solutions with pH values significantly lower than 6.0 and negatively charged at pH values significantly higher than 6.0.

### 3.8 Wettability

The wettability or hydrophilic/hydrophobic character of a material is evaluated based on the contact angle (θ) formed between the surface of the material and a water droplet. A high contact angle (θ > 90°) indicates a hydrophobic surface, while a low contact angle (θ < 90°) suggests a hydrophilic surface. The results for each material are presented in Figure S1, facilitating a comparison of their surface properties. The measured values were 56° for CS/PPy indicating hydrophilic character and 108° for CS/PPy/GA suggesting a hydrophobic character. The presence of hydroxyl (−OH) groups in CS and amine (−NH_2_) groups in PPy can promote hydrogen bonding with water molecules, leading to increased hydrophilicity. In addition, this observation for CS/PPy/GA aligns with the general trend that cross-linking a material can reduce its water affinity.^18^

### 3.9 DLS

DLS was employed to measure the hydrodynamic diameter of suspended particles. DLS measurements were performed on CS/PPy and CS/PPy/GA dispersed in Britton-Robinson buffer at pH 7.4, 6.0, and 4.5, simulating the pH of blood, tumor, and lysosomal environments, respectively. The particle size distribution for the materials CS/PPy, CS/PPy/GA at different pHs has been obtained. To facilitate a comparison of the materials’ behavior at different pHs, Figure 4A presents the average particle size for CS/PPy and CS/PPy/GA. The average particle size of CS/PPy nanoparticles gradually decreased with increasing pH, from 117.00 ± 5.90 nm at pH 4.5 to 112 ± 5.6 nm at pH 6.0 and to 104.00 ± 5.20 nm at pH 7.4. These observations are corroborated by TEM analysis, which revealed particle sizes in the range of 78–184 nm, further supporting the DLS results. In contrast to CS/PPy, the average particle size of CS/PPy/GA nanoparticles increased with increasing pH. The sizes were 842 ± 42.1 nm at pH 4.5, 944 ± 47.2 nm at pH 6.0, and 1000 ± 50.0 nm at pH 7.4. The DLS measurements for CS/PPy/GA show significantly larger sizes compared to the particle dimensions observed by TEM. This discrepancy can likely be attributed to two factors: (i) the swelling behavior of CS/PPy/GA in aqueous solution; (ii) a possible interference of the dispersant used on the measured hydrodynamic diameter by DLS. Additionally, this difference highlights the complementary nature of these techniques. DLS provides information on the average hydrodynamic diameter in solution, which can be influenced by factors like solvated polymer chains or aggregates, while TEM offers direct visualization of individual particle morphology.

**Figure 4:**
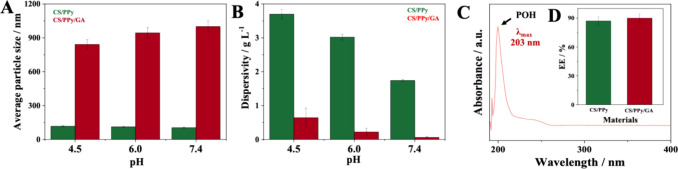
(A) Average particle size, (B) dispersivity test and (C) UV–vis spectrum of POH in methanol; and (D) encapsulation efficiency (EE%) of POH by CS/PPy (green) and CS/PPy/GA (red) at different pHs (4.5, 6.0, and 7.4).

Nanoparticles are formed through aggregation between polymer chains. These interactions can be through covalent bonds or weaker intermolecular forces. In the case of CS/PPy nanoparticles, these are formed by weaker electrostatic forces; there is no formation of covalent bonds, only electrostatic interactions. These particles are formed after the dispersion of the material in an aqueous medium, and their size is influenced by the pH of the medium. As observed for the CS/PPy material, at lower pH values, a larger particle size is observed. This is due to the protonation of the CS amino groups, which causes a certain repulsion between the chains due to the repulsion between the positively charged amino groups, as well as a greater interaction with water molecules, which does not occur at higher pH values, since under these conditions the amino groups are deprotonated, resulting in greater aggregation of the material. The CS/PPy/GA material also forms particles through electrostatic interactions between the chains. However, the larger particle sizes compared to the CS/PPy material are due to the action of the cross-linking agent used. This covalently joins one CS chain to another through the formation of an imine group, resulting from the reaction between the amino group of the CS and the aldehyde of the glutaraldehyde. This results in the aggregation of a greater number of chains and, consequently, a larger particle size. Contrary to what is observed with the CS/PPy material, a decrease in particle size is observed at acidic pH. This is due to the possible hydrolysis of the imine groups, which decreases the rate of cross-linking between the chains and, consequently, there is a decrease in their sizes.

DLS measurements revealed that the average particle size of the CS/PPy and CS/PPy/GA was on a nanometric scale. However, according to literature reports, a nano size for polymeric particles in the range of 10–1000 nm is acceptable.^26–28^ For intravenous administration of targeted macromolecular drugs for cancer, nanocarriers ideally exhibit a size above 100 nm. Smaller particles (<100 nm) can potentially extravasate (leak) from the vasculature in healthy tissues and be rapidly cleared by the kidneys, reducing their therapeutic efficacy. To optimize tumor accumulation and therapeutic effect, these targeted nanoparticles should ideally possess a size between 100 and 500 nm. Particles larger than 500 nm are more susceptible to clearance by the mononuclear phagocytic system, which includes macrophages that eliminate foreign substances.^29–33^

### 3.10 Dispersivity Tests

To assess the dispersion behavior of CS/PPy and CS/PPy/GA under conditions mimicking biological environments, a dispersivity test was employed. These environments included: Lysosomal environment of cancer cells (simulated by pH 4.5), tumor environment (simulated by pH 6.0), bloodstream environment (simulated by pH 7.4). The results of this test will indicate which material exhibits superior dispersivity in these environments, potentially impacting their performance as drug delivery systems. Figure 4B presents the results of the dispersivity test, indicating how much CS/PPy and CS/PPy/GA remained dispersed in the supernatant after centrifugation under various pH conditions. These findings reveal that CS/PPy/GA exhibited significantly lower dispersivity compared to CS/PPy across all simulated biological environments (pH 4.5, 6.0, and 7.4). The data suggests that CS/PPy/GA may have dispersed material up to six times less than CS/PPy under these conditions. The observed lower dispersivity of CS/PPy/GA compared to CS/PPy might be attributed to the cross-linking effect of GA. This cross-linking could potentially immobilize the ionizable groups within CS/PPy/GA, hindering their interaction with the aqueous environments (pH 4.5, 6.0, and 7.4) and reducing dispersive forces. The wettability test results, potentially indicating a higher contact angle close to 90° for CS/PPy/GA, could further support this hypothesis of increased hydrophobicity. The materials exhibited enhanced dispersivity at pH 4.5 (acidic environment). This improved dispersion likely arises from a higher concentration of protonated amino groups in the CS component of the nanoparticles. Protonation increases the positive charge density on the particle surface, leading to stronger electrostatic repulsions between particles, which helps prevent aggregation and promotes better dispersion in the aqueous medium. At pH 7.4 (basic environment), both CS/PPy and CS/PPy/GA displayed lower dispersivity. This observation aligns with the expected behavior based on their pH_PZC_. Since the pH is higher than the pH_PZC_, the amino groups the CS are likely deprotonated, resulting in a reduced overall positive charge density on the nanoparticles. This decrease in electrostatic repulsion between particles can favor aggregation and contribute to the observed lower dispersivity. This can lead to increased hydrophobicity of the materials.

While CS/PPy/GA exhibits good dispersivity across a range of pH values, its potential application as a nanocarrier for intravenous drug delivery might be limited by particle size. Nanoparticles exceeding 500 nm in diameter are susceptible to excreted by the body through the phagocytosis process. This rapid clearance can significantly reduce circulation time and hinder the ability of the nanoparticles to reach their target site within the body. Studies by Ruan et al. (2015) using animal models suggest that the pore size of tumor vasculature can range from 10 to 2000 nm in diameter.^200^ Considering that only very large nanoparticles (>2 μm) are likely to significantly obstruct blood vessels with capillary diameters around 5 μm, encapsulation and controlled release studies were performed with both materials. These studies aimed to evaluate their efficiency as drug delivery systems.^30,34^

### 3.11 EE%

To determine the wavelength of maximum absorption (λ_max_) for POH, a standard sample was analyzed using UV–vis spectroscopy in the range of 190–400 nm. The results are presented in Figure 4C. The study investigated the potential of both synthesized materials (CS/PPy and CS/PPy/GA) as nanocarriers for POH delivery. The amount of unencapsulated POH remaining in the supernatant after encapsulation with each material was quantified. This quantification was achieved by comparing the supernatant’s absorbance values at λ_max_ with a calibration curve established using the POH standard (Figure S1). The EE% for each material was calculated using eq 1. The calculated EE% values are presented in Figure 4D. The EE% of both materials were comparable, with values of 87% for CS/PPy and 90% for CS/PPy/GA. However, CS/PPy/GA exhibited the highest EE% for POH. The observed higher encapsulation efficiency of CS/PPy/GA might be attributed to the cross-linking effect of GA. This cross-linking could potentially create a more intricate network within the CS/PPy matrix, offering more suitable sites for POH entrapment and an increase in its contact surface.

In their study, Marson et al. investigated polymeric nanoparticles made from poly­(lactic-*co*-glycolic acid) for encapsulating the POH. They achieved an EE% around 74%, using HPLC for quantification.^26^ Building on their previous work, Marson et al. explored a new material for POH encapsulation. They developed diblock polymeric nanoparticles composed of poly­(lactic acid) and poly­(ethylene glycol). This material achieved an EE% for POH in the range of 63–68%. HPLC was again employed for quantification.^34^ Penteado et al. investigated the use of poly­(ε-caprolactone) nanocapsules coated with chitosan for POH encapsulation. This system achieved an EE% of 56%. HPLC was employed for the quantification analysis.^35^ The EE% achieved for POH using the synthesized materials in this study are comparable to or higher than those reported in the literature.

### 3.12 In Vitro Release Profile

Following the encapsulation of POH by the synthesized materials and evaluation of their encapsulation efficiencies, in vitro release studies were performed. These tests monitored the release of POH over 48 h at 37 °C in solutions with pH values of 4.5, 6.0, and 7.4. These pH values simulate the environments of lysosomes in cancer cells (pH 4.5), tumor microenvironments (pH 6.0), and blood (pH 7.4), respectively. The amount of POH released from the materials at different time points was quantified using UV–vis spectrophotometry by calibration curves showed in Figure S2. The absorbance of each sample was measured at the wavelength of maximum absorption for POH (λ_max_ = 203 nm, as established earlier). The in vitro release profiles of POH encapsulated within the CS/PPy and CS/PPy/GA are presented in Figure 5. Table S1 presents detailed data on the percentage (%) of POH released from CS/PPy and CS/PPy/GA at various time points (up to 48 h) and under the different pH conditions (4.5, 6.0, and 7.4).

**Figure 5:**
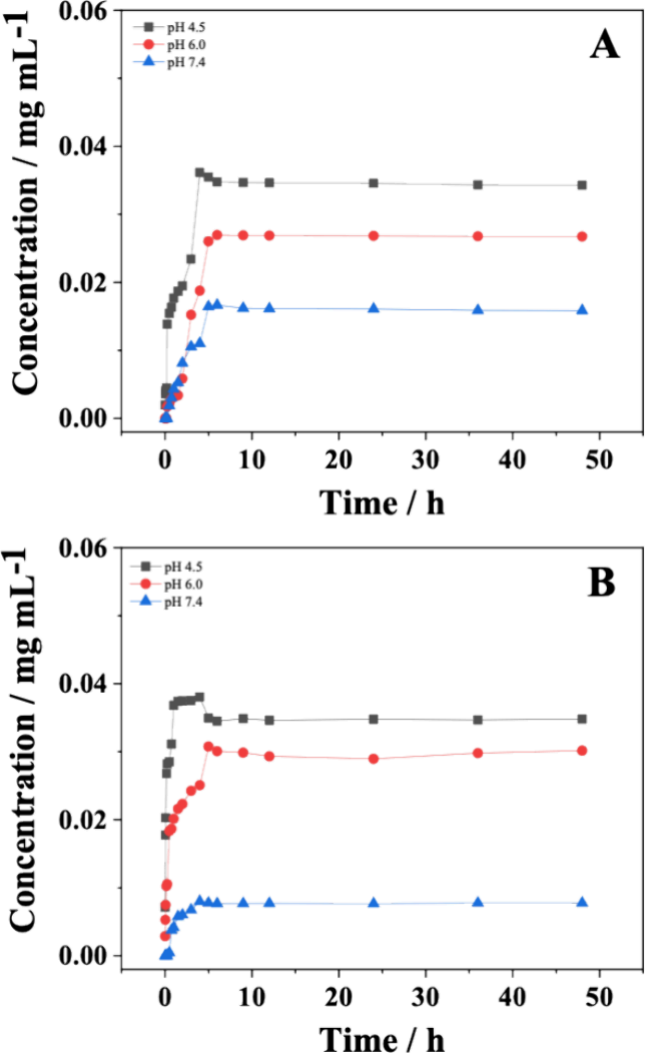
Release profiles of POH encapsulated by materials (A) CS/PPy and (B) CS/PPy/GA at pHs 4.5, 6.0, and 7.4 in Britton-Robinson buffer to simulate the pH of the lysosomal, tumor, and blood environments, respectively.

The POH in vitro release profiles showed a pH dependence. This observation implies that the environment’s acidity may have an impact on the materials’ release behavior. In the acidic environment (pH 4.5), both materials showed a higher rate of POH release than in the neutral (pH 7.4) and slightly basic (pH 6.0) environments. Since POH has low solubility in aqueous media and has hydrophobic characteristics. It is incorporated by the particles through its interaction with the hydrophobic regions formed inside the nanoparticles. Therefore, its greater release in an acidic pH medium is due to the reduction of these regions inside the nanoparticles. Since the protonation of the amino groups causes an expansion between the chains and also greater hydration, exposing the POH to the medium. In these highly acidic media, pH-sensitive nanocarriers provide drug release into the tumor interstitium, causing its inhibition. For CS/PPy and CS/PPy/GA, the maximum release of POH was observed after 4 h, reaching approximately 12 and 10%, respectively.

In the buffer solution with pH 6.0, CS/PPy and CS/PPy/GA exhibited similar POH release profiles. After 5 h, approximately 8.5% of the POH was released from CS/PPy, while CS/PPy/GA released around 9.0% after 6 h. As expected, CS/PPy and CS/PPy/GA displayed a significantly lower release rate of POH in the pH 7.4 buffer solution. After 4 and 6 h, respectively, only around 2 and 5% of the encapsulated POH was released from CS/PPy and CS/PPy/GA. The slow release of POH observed at pH 7.4 (which mimics healthy blood plasma) by CS/PPy and CS/PPy/GA suggests these materials could potentially minimize premature release during systemic circulation. This could reduce potential side effects on healthy tissues and improve the therapeutic efficacy by ensuring targeted delivery to the site of action. This slow release of POH at pH 7.4 might be attributed also to a stronger interaction between the encapsulated POH and the CS/PPy and CS/PPy/GA under neutral/basic conditions.

The development of pH-responsive polymeric materials for drug encapsulation and controlled release in cancer treatment has been an active area of research, with numerous studies reported in the literature. Mahmoodzadeh et al. designed a chitosan-based nanogel for controlled drug delivery. This nanogel was engineered by grafting 2-hydroxyethyl methacrylate and maleic acid onto CS, followed by cross-linking with *N,N’*-bis­(acryloyl) cystamine. The researchers evaluated its potential for doxorubicin release in cancerous tissue. The release rate of the encapsulated drug was significantly higher at the acidic pH (5.3) of cancerous tissue (82%) compared to the neutral pH (7.4) of blood plasma (71%).^36^ Another study explored a nanogel based on CS as a carrier for polyoxometalates (POMs) in breast cancer treatment. This research achieved a controlled release of approximately 30% of the POMs from of the CS matrix.^37^

Other researchers investigated the use of polymeric micellar systems formed from amphiphilic *N*-alkyl-*N*-trimethyl chitosan derivatives for controlled release of 10-hydroxycamptothecin, a hydrophobic anticancer drug. The observed EE% of these materials varied considerably, from 7.1 to 56.1%. This variation likely stems from the ratio of drug to polymer used during their preparation. The researchers compared the in vitro release profiles of the polymeric micelles loaded with 10-hydroxycamptothecin to a commercially available lyophilized powder of the same drug. The polymeric micelles exhibited a sustained release profile for 10-hydroxycamptothecin. It took approximately 110 h for 50% of the loaded drug to be released, and around 620 h to reach the maximum release. This observation suggests the absence of a significant burst release, which is desirable for controlled drug delivery as it minimizes initial high doses and ensures a more prolonged therapeutic effect. In contrast, the commercially available lyophilized powder formulation of 10-hydroxycamptothecin exhibited a significantly faster release. Roughly 70% of the drug was released within just 11 h, and near complete release was observed within 34.5 h. These findings suggest that *N*-alkyl-*N*-trimethyl CS derivatives and similar materials hold promise for controlled delivery of hydrophobic camptothecin drugs. By enabling sustained release, this approach has the potential to improve the safety, efficacy, stability, and pharmacokinetic properties of these drugs.^38^

Farazuddin et al. investigated the use of polylactic glycolic acid (PLGA) microparticles as carriers for POH. Their study achieved an entrapment efficiency of 42.4%, indicating a successful encapsulation of the therapeutic agent within the microparticles. It was also observed a biphasic release profile for POH from the PLGA microparticles. The system exhibited an initial burst release followed by a sustained release phase over an extended period. The initial release of POH from the PLGA microparticles exhibited a slow-release pattern. Only around 12% of the encapsulated POH was released within the first 18 h. The release rate then increased slightly, with an additional 17% released in the following 12 h, reaching a cumulative release of approximately 22% by the 72-h mark. The PLGA microparticles in this study exhibited a cumulative release of approximately 30% of the entrapped drug over a period of 7 days (168 h).^39^ Penteado et al. explored the use of CS-coated poly­(ε-caprolactone) nanocapsules as a delivery system for POH. It was observed a biphasic release profile for POH. There was an initial burst release of approximately 16% of the encapsulated drug within the first 24 h. This was followed by a sustained release phase, where 26% was released over the next 168 h (7 days), reaching a cumulative release of around 42% by the end of the study.^35^ In view of the above, it can be observed our results demonstrate that the synthesized materials exhibited a low release profile for POH. The observed slow release of POH could be attributed to its lipophilic nature. As a lipophilic compound, POH likely exhibits stronger interactions with the hydrophobic interior of the synthesized materials compared to the aqueous environment. This preferential interaction within the carrier matrix could delay its release into the surrounding water.

In this work it was investigated the release kinetics of POH by the CS/PPy and CS/PPy/GA at different pH values. To understand the release mechanisms, was fitted the release data to kinetic models, including the Zero-Order, First-Order, Higuchi, and Korsmeyer-Peppas models. This analysis aimed to identify the dominant mechanism governing POH release from these materials. The coefficient of determination (*R* ^2^) values, presented in Table 2, were used to identify the most suitable model for describing the release kinetics of POH from each material. A higher *R* ^2^ value indicates a better fit between the model and the experimental data.

**Table 2:** *R* ^2^ Values of the Evaluated Release Models

		zero order		first order		Higuchi		Korsmeyer-Peppas		
materials		*R* ^2^	*K*_0_	*R* ^2^	*K*_1_	*R* ^2^	*K*_H_	*R* ^2^	*K*	*n*
CS/PPy	pH 4.5	0.7586	1.3646	0.7666	0.0147	0.9062	4.4654	0.9382	5.4412	0.4808
	pH 6.0	0.9036	1.3052	0.9048	0.0135	0.9066	3.9144	0.9203	1.4226	0.9429
	pH 7.4	0.8756	0.7765	0.8778	0.0078	0.9541	2.4270	0.9570	1.3566	0.7833
Cs/PPy/GA	pH 4.5	0.5106	1.5005	0.5199	0.0161	0.7244	3.7656	0.8176	9.1685	0.2548
	pH 6.0	0.7542	1.0853	0.7629	0.0112	0.9078	3.0605	0.9527	4.9079	0.3771
	pH 7.4	0.8180	0.5060	0.8214	0.0101	0.9224	1.2634	0.6385	1.3807	0.9206

Analyzing POH release data through kinetic models is crucial for understanding the relationship between the release profile and the potential bioavailability of the encapsulated drug. By analysis of the *R* ^2^ values for CS/PPy revealed that the Korsmeyer-Peppas model provided the best fit for the POH release kinetics at all three investigated pH. This suggests that the release mechanism for POH from CS/PPy is likely a combination of diffusion and erosion. The analysis of the release exponent (n) for the CS/PPy material further suggests a non-Fickian release mechanism (anomalous case) at pH 4.5 and 7.4. In this mechanism, the release rate is controlled by a combination of diffusion and polymer chain relaxation. This indicates that the release behavior is more complex than simple diffusion and may involve some erosion of the polymer matrix. At pH 6.0, the release profile of POH from CS/PPy suggests a non-Fickian release mechanism (Super case II). In this mechanism, the release is likely governed by the relaxation and/or degradation of the CS/PPy material, rather than simple diffusion. At pH 4.5, the protonation of the polymer chains in CS/PPy likely leads to electrostatic repulsion between the positively charged groups. This repulsion can cause expansion of the polymeric network, potentially influencing the release of POH. An expanded polymer network can facilitate the diffusion of water into the CS/PPy matrix. This enhanced water penetration might then promote the dissolution and release of POH due to its hydrophilic nature. At pHs 6.0 and 7.4, it can be considered that there is less diffusion of water from the medium and, therefore, a lower rate of POH release.

Like CS/PPy, the analysis of *R* ^2^ values suggests that the Korsmeyer-Peppas model also provides the best fit for describing the POH release kinetics from CS/PPy/GA at pH 4.5 and 6.0. The release exponent (*n*) values for CS/PPy/GA at pH 4.5 (*n* = 0.2548) and pH 6.0 (*n* = 0.3771) are lower than 0.45. This suggests a mechanism from Fickian diffusion. While these values indicate some contribution from diffusion, they might also suggest involvement of other mechanisms such as polymer relaxation or erosion. At pH 7.4, the Higuchi model exhibited the highest *R* ^2^ value, suggesting that diffusion is the dominant mechanism controlling POH release from CS/PPy/GA. The presence of GA cross-linking in CS/PPy/GA might enhance diffusion by creating a more open and interconnected network within the material. This increased accessibility could facilitate the movement of water molecules into the matrix, potentially promoting the diffusion of POH for release.

## 4 Comparison with Other Nanocarriers

Nanocarrier systems have emerged as a promising strategy for therapeutic drug delivery. Numerous studies have documented their potential to improve targeting specificity, and control drug release. This has generated significant interest in their application for various diseases. Table 3 compares the EE% of POH for CS/PPy and CS/PPy/GA, alongside other relevant parameters, with previously reported CS-based nanocarrier systems designed for targeted drug delivery in cancer treatment. It is possible to observe that the synthesized materials exhibit high EE% for POH, comparable to previously reported systems. This suggests their potential for efficient drug loading in therapeutic applications. The observed drug release rates for our CS-based materials differ from the findings reported in some previous studies utilizing pH-responsive polymers. This highlights the importance of considering factors such as material composition and environmental conditions when designing pH-responsive drug delivery systems in each study. The results suggest that the synthesized materials exhibited high affinity for POH. This strong interaction between the materials and POH likely contributes to the low release rate observed in this study. However, it can be considered that the materials were promising and reproducible, since it was possible to obtain results like studies already reported.

**Table 3:** Comparison between CS-Based Anticancer Drug Delivery Nanocarrier Systems^a^

nanocarriers	drug	EE%	release time/h	quantity released/%	kinetic model	ref.
CS/PPy	POH	87	48	12% in pH 4.5	Korsmeyer-Peppas	this work
				8.5% in pH 6.0		
				2.0% in pH 7.4		
CS/PPy/GA		90		10% in pH 4.5	Korsmeyer-Peppas pH 4.5 and 6.0 Higuchi pH 7.4	
				9.0% in pH 6.0		
				5.0% in pH 7.4		
CS/HNT/CNT	curcumin	88	96	92	Baker for pH 5.4 Higuchi for pH 7.4	^40^
F-PEG-HTCC-coated SLN	paclitaxel	99	72	50	-	^41^
O-CMCS/*n*-ZnO	curcumin	74	144	35% in pH 7.4	-	^42^
				48% in pH 4.5		
CS/DEX/CS	paclitaxel	66.3	150	51.9% in pH 5.67	two phases Higuchi first order	^43^
				40.0% in pH 6.58		
				32.1% in pH 7.4		
	5-Fu-5-fluorouracil	75.2		99.4% in pH 5.67	Higuchi	
				96.2% in pH 6.58		
				87.0% in pH 7.40		
ACNPs	amygdalin	90	10	70.5% in pH 3.1	-	^44^
				81.9% in pH 5.0		
				86.0% in pH 7.4		
SA/CTS NPs	salicylic acid	84	48	68% in pH 5.5	Korsmeyer-Peppas	^45^
				31% in pH 7.4		
COOH-Chi-MSNs	doxorrubicin	56.72	144	80.2% in pH 5.5	-	^46^
				55.6% in pH 6.5		
				37.9% in pH 7.4		
GO–CH–Ma	*Ulva lactua*	88	70	20% in pH 7.4	Korsmeyer-Peppas	^47^
				70% in pH 5.3		
MIL-101/GA-CS	doxorrubicin	74.47	72	2.74% in pH 7.4	-	^48^
				89.18% in pH 5.5		
CMCS-*g*-gly	vincristine sulfate	56.97–71.91	120	75.50% in pH 5	first order for pH 5 Korsmeyer-Peppas for pH 7.4	^49^
				5.94% in pH 7.4		
CS-*g*-gly		72.28–89.97		82.27% in pH 5	Korsmeyer-Peppas for pH 5 first Order for pH 7.4	
				25.83% in pH 7.4		
Cap-ALG NPs@PCLCS NFs	capsaicin	98.7	500	100	Korsmeyer-Peppas	^50^

a CS/HNT/CNT, chitosan/halloysite nanocarrier modified by carbon nanotube; F-PEG-HTCC-coated SLN, folate-poly­(ethylene glycol)-*N*-[(2-hydroxy-3-trimethylammonium) propyl] chitosan coated with solid lipid nanoparticles; O-CMCS/n-ZnO, O-carboxymethyl chitosan with nanostructured zinc oxide; CS/DEX/CS, chitosan/dextran sulfate/chitosan; ACNPs, alginate–chitosan nanoparticles; SA/CTS NPs, Salicylic acid-loaded chitosan nanoparticles; COOH-Chi-MSNs,, carboxylated chitosan mesopouros sílica nanoparticles, GO–CH–Ma, chitosan and d-manose-functionalized graphene oxide; MIL-101/GA-CS, Metal–organic framework decorated with glycyrrhetinic acid conjugated chitosan; CMCS-*g*-gly, carboxymethyl chitosan-*graft*-glycerol; CS-*g*-gly, chitosan-*graft*-glycerol; Cap-ALG NPs@PCLCS NFs, Capsaicin-loaded alginate nanoparticles embedded polycaprolactone-chitosan nanofibers.

## 5 Conclusions

This study successfully synthesized nanometric and pH-responsive materials by coupling the natural polymer CS with the synthetic polymer PPy. The resulting materials, CS/PPy and CS/PPy/GA, exhibited pH-dependent solubility and size. FTIR analysis of the synthesized materials revealed characteristic bands corresponding to functional groups present in both CS and PPy. This confirms the successful formation of composite materials where CS and PPy are chemically linked. The high EE% values observed for both synthesized materials suggest a strong affinity for POH. The POH release profiles clearly demonstrated the significant influence of medium pH on POH release. This pH-responsive release behavior highlights the potential for these materials to be targeted drug delivery systems. The synthesized materials exhibited faster and more extensive POH release under acidic conditions compared to neutral or alkaline environments. This release mechanism minimizes potential damage to healthy tissues by targeting POH release to the acidic environment often found within tumor cells. The CS/PPy exhibited a maximum release of approximately 12% of the loaded POH within the first 4 h. The Korsmeyer-Peppas model provided the best fit for describing the release kinetics. Like CS/PPy, CS/PPy/GA achieved a maximum release of around 10% of the loaded POH within 4 h. The Korsmeyer-Peppas model provided the best fit for describing the release kinetics at pH 4.5 and 6.0. Higuchi’s model better described the release behavior at pH 7.4, indicating a possible shift in the dominant release mechanism under more neutral conditions.

## Supplementary Material



## Notes

Additional results regarding the contact angle measurements for wettability of the materials; particle size distribution of CS/PPy and CS/PPy/GA at pHs 4.5, 6.0, and 7.5; analytical curves prepared at different concentrations of POH in methanol to obtain encapsulation efficiency of POH by materials CS/PPy and CS/PPy/GA, amount of POH release by CS/PPy at different media, amount of POH release by material CS/PPy/GA at different media; and cumulative percentage of POH released by different materials (PDF)
