# Supplementary material for: Controlled Release of Perillyl Alcohol via pH-Responsive Chitosan-Polypyrrole Nanocarriers
Source: ACS Omega. 2025 Jun 25;10(26):28466–77. doi: 10.1021/acsomega.5c04817 (PMC12242680; doi:10.1021/acsomega.5c04817)
Supplement: Supplementary file 1 [file ao5c04817_si_001.pdf]

Controlled Release of Perillyl Alcohol via pH-Responsive Chitosan-Polypyrrole Nanocarriers

Marcella Matos Cordeiro Borges[a], Stephanne Yonara Barbosa de Carvalho[a], Keyller Bastos Borges[a]* and Luiz Gustavo de Lima Guimarães[a]*

[a] Departamento de Ciências Naturais, Universidade Federal de São João del-Rei, Campus Dom Bosco, Praça Dom Helvécio 74, Fábricas, 36301-160, São João del-Rei, Minas Gerais, Brazil

*Corresponding author:
Prof. Luiz Gustavo de Lima Guimarães, PhD, Departamento de Ciências Naturais, Universidade Federal de São João del-Rei, Campus Dom Bosco, Praça Dom Helvécio 74, Fábricas, 36301-160, São João del-Rei, Minas Gerais, Brazil
*e-mail: lguimaraes@ufsj.edu.br; Tel.: +55 32 3379–5163

Prof. Keyller Bastos Borges, PhD, Departamento de Ciências Naturais, Universidade Federal de São João del-Rei, Campus Dom Bosco, Praça Dom Helvécio 74, Fábricas, 36301-160, São João del-Rei, Minas Gerais, Brazil
*e-mail: keyller@ufsj.edu.br; Tel.: +55 32 3379–5163

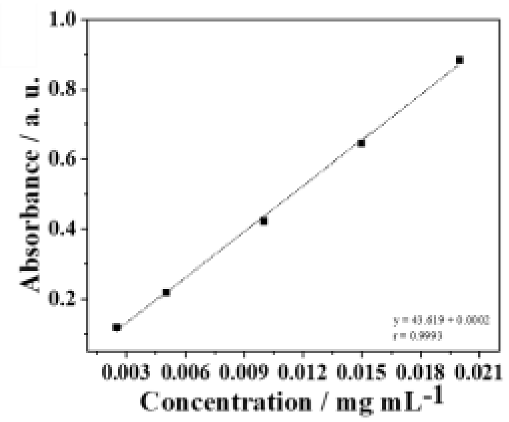

Figure S1. Analytical curve prepared at different concentrations of POH in methanol, to obtain EE% of POH by materials CS/PPy and CS/PPy/GA.

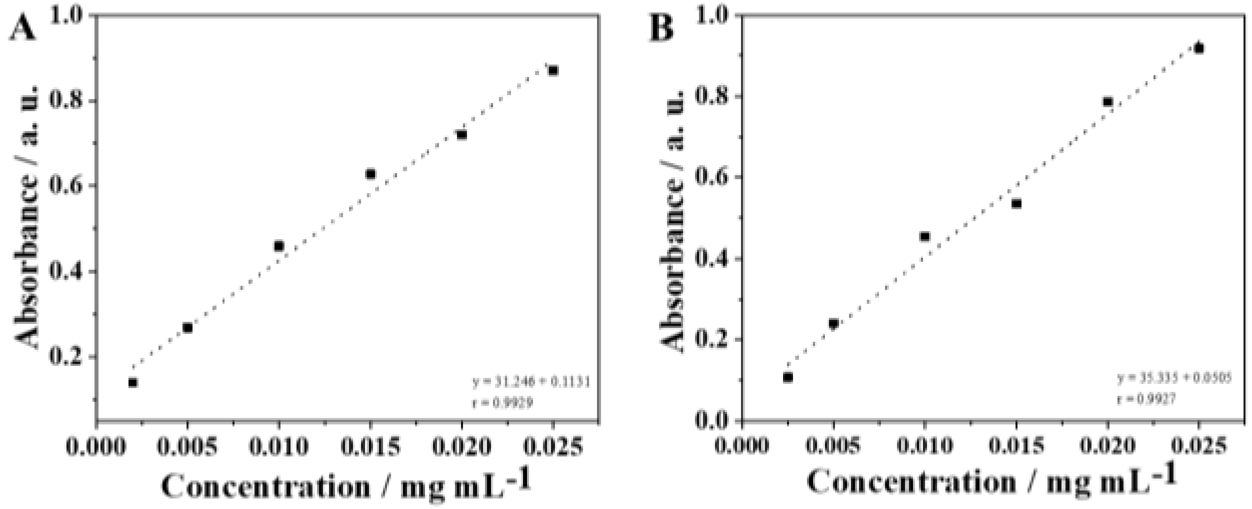

Fig. S2. Analytical curves prepared at different concentrations of POH in methanol, to obtain: (A) amount of POH released by the CS/PPy material in the different pH; (B) amount of POH released by the materials CS/PPy/GA.

Table

Table S1. Cumulative percentage of POH released by different materials.

Time / h	% Cumulative of POH release		
	**pH**	**CS/PPy**	**CS/PPy/GA**
0.017	4.5	0.68	1.97
	6.0	0	0.79
	7.4	0	0
0.05	4.5	1.24	4.93
	6.0	0	1.47
	7.4	0	0
0.08	4.5	1.40	5.63
	6.0	0	2.07
	7.4	0	0
0.17	4.5	1.54	7.44
	6.0	0	2.84
	7.4	0	0
0.25	4.5	4.77	7.84
	6.0	0.59	2.92
	7.4	0	0
0.5	4.5	5.33	7.92
	6.0	0.74	5.09
	7.4	0.65	0.13
0.75	4.5	5.64	8.65
	6.0	0.98	5.18
	7.4	1.04	1.04
1	4.5	6.10	10.23
	6.0	1.13	5.60
	7.4	1.50	1.16
1.5	4.5	6.44	10.39
	6.0	1.17	6.00
	7.4	1.82	1.60
2	4.5	6.72	10.41
	6.0	2.02	6.20
	7.4	2.81	1.67
3	4.5	8.07	10.42
	6.0	5.25	6.74
	7.4	3.64	1.88
4	4.5	12.47	10.56
	6.0	6.47	6.96
	7.4	3.80	2.22
5	4.5	12.23	9.71
	6.0	8.98	8.54
	7.4	5.67	2.16
6	4.5	11.99	9.58
	6.0	9.30	8.36
	7.4	5.75	2.13
9	4.5	11.96	9.68
	6.0	9.28	8.29
	7.4	5.59	2.13
12	4.5	11.95	9.61
	6.0	9.27	8.14
	7.4	5.56	2.14
24	4.5	11.91	9.66
	6.0	9.26	8.04
	7.4	5.55	2.13
36	4.5	11.84	9.63
	6.0	9.23	8.28
	7.4	5.48	2.16
48	4.5	11.83	9.67
	6.0	9.22	8.38
	7.4	5.47	2.15
